# Neuro-Musculoskeletal Mapping for Man-Machine Interfacing

**DOI:** 10.1038/s41598-020-62773-7

**Published:** 2020-04-02

**Authors:** Tamas Kapelner, Massimo Sartori, Francesco Negro, Dario Farina

**Affiliations:** 1 Orthopaedic Surgery and Plastic Surgery — Research Department of Neurorehabilitation Systems, Clinic for Trauma Surgery, University Medical Center Göttingen, Göttingen, 37075 Germany; 2 Department of Biomechanical Engineering, TechMed Centre, University of Twente, Enschede, Netherlands; 3 Department of Clinical and Experimental Sciences, Research Centre for Neuromuscular Function and Adapted Physical Activity “Teresa Camplani”, Università degli Studi di Brescia, Brescia, Italy; 4 Department of Bioengineering, Imperial College London, SW7 2AZ London, UK

**Keywords:** Quality of life, Biomedical engineering

## Abstract

We propose a myoelectric control method based on neural data regression and musculoskeletal modeling. This paradigm uses the timings of motor neuron discharges decoded by high-density surface electromyogram (HD-EMG) decomposition to estimate muscle excitations. The muscle excitations are then mapped into the kinematics of the wrist joint using forward dynamics. The offline tracking performance of the proposed method was superior to that of state-of-the-art myoelectric regression methods based on artificial neural networks in two amputees and in four out of six intact-bodied subjects. In addition to joint kinematics, the proposed data-driven model-based approach also estimated several biomechanical variables in a full feed-forward manner that could potentially be useful in supporting the rehabilitation and training process. These results indicate that using a full forward dynamics musculoskeletal model directly driven by motor neuron activity is a promising approach in rehabilitation and prosthetics to model the series of transformations from muscle excitation to resulting joint function.

## Introduction

State-of-the-art upper limb prostheses receive control commands from the user through a myoelectric interface. With this interface, the muscle fiber electrical activity in the residual limb is detected by recording surface electromyograms (sEMG), which are used to predict the user’s motor intent and to control a prosthesis accordingly. Most devices use two electrode systems over an antagonist muscle pair to proportionally control one degree of freedom (DoF), e.g. hand open/close^1^. Actuating more than one DoF requires a switching mechanism that leads to a slow, sequential, and unintuitive control of a maximum of two DoFs^2^. This limited functionality in conjunction with the required extensive training leads to high abandonment rates of these prosthetic devices^3,4^.

To overcome this limitation and to provide natural control, classification methods have been proposed to estimate the movement of the wrist and hand from EMG features^5^. Similarly, regression methods have been developed to continuously estimate kinematic variables (i.e. angular position or velocity of the joints), so that the user can actuate multiple DoFs concurrently^6,7^. Both classification and regression approaches outperform conventional control methods in complex tasks, in terms of both speed and accuracy, providing a promising direction for advanced myoelectric control^8–11^.

Further improvement of regression methods might be facilitated by extracting the neural information embedded in the EMG. The neural drive to a muscle is the ensemble of discharges of its innervating motor neurons^12,13^. Recent advances in the decomposition of the surface EMG allows for accurate decoding of the neural drive^14–16^.

Previously, we demonstrated that it is possible to extract neural information from high-density, multi-channel EMG used for myoelectric control in intact-bodied subjects and transradial amputees and in patients following targeted muscle reinnervation^17–19^. The application of such information for control showed that neural information outperforms conventional EMG features^20^. However, current machine learning approaches for regression estimate kinematic information using generic model-free learning algorithms, reducing the underlying neuro-musculoskeletal processes involved in the motor task to a single regression function. Such approximations might fail to capture the complexity of the neuro-mechanical transformations, limiting the intuitiveness and robustness of these methods^6,21–23^.

A promising alternative is to employ a biomechanical modeling approach as we have recently demonstrated^24–26^. In this context, a neuro-musculoskeletal (NMS) model is used to translate neuromuscular activity into the resulting mechanical function^26,27^. In the context of prosthesis control, this model would realize a mapping from the EMG into prosthesis commands^24^. Although using global EMG features as an input for such models proved to be feasible^25^, the estimation of the full neuro-mechanical transformation chain may be further optimized by decoding the neural drive to muscles and using it as input for a muscolo-skeletal simulation^28^.

In this study, we propose a control strategy for amputees based on NMS modeling^24,25^ with the addition of motoneuron activity as input. The proposed control strategy would combine a mapping of neural activity into muscle excitations, and a musculoskeletal mapping of the muscle activations into wrist kinematics, in order to model the whole neuro-mechanical transformation chain. We present a proof of concept of this novel prosthesis control method by extracting neural information from high-density surface EMG using blind source separation, mapping the neural information to muscle excitations using robust linear regression, and predicting joint angles from estimated muscle excitations using biomechanical modeling. We demonstrate the feasibility of offline wrist kinematics prediction with the proposed method in single DoF tasks and show that it outperforms state of the art regression methods. We show for the first time a successful application of a musculoskeletal model driven by neural information for prosthesis control strategies in transradial amputees.

## Results

**Figure 1:**
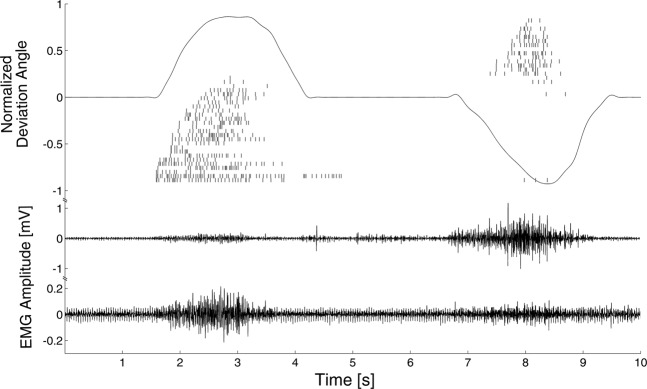
Representative example of the decomposition results, the captured kinematics and the raw recorded EMG.

The decomposition identified on average 19 ± 7 motor units per DoF, corresponding to 63 ± 16 motor units per subject (Fig. 1, Table 1). The results of the assignment of these spike trains to muscles by the NMS model, as described in Methods, is detailed in Table 2.

**Table 1:** The number of decomposed spike trains per DoF for each subject.

	DoF_1_	DoF_2_	DoF_3_	DoF_12_	DoF_13_	DoF_23_	DoF_123_	Total
A1	16	16	30	0	0	3	0	65
A2	7	17	9	3	0	4	1	40
A3	23	21	11	2	2	1	0	60
A4	18	30	22	4	0	0	0	74
A5	4	12	18	3	1	4	0	42
A6	19	29	25	1	1	5	0	80
D1	23	29	24	2	3	6	1	87
D2	17	24	14	1	0	0	0	56

DoF1 was flexion/extension, DoF2 was radial/ulnar deviation, and DoF3 was Pronation/Supination. Columns with more than one index contain spike trains detected in more than one DoF, e.g. DoF_123_ contains spike trains of motor units active during all DoFs.

**Table 2:** The average number of spike trains assigned to each muscle in the NMS model, for each subject.

	SUP	ECRL	ECU	FCR	FCU	PT	Not assigned
A1	14 ± 0 (0)	12 ± 1 (9 ± 3)	16 ± 1 (16 ± 1)	11 ± 3 (11 ± 3)	18 ± 0 (18 ± 0)	13 ± 0 (0)	5 ± 1
A2	5 ± 1 (1 ± 0)	18 ± 1 (14 ± 1)	12 ± 1 (12 ± 1)	12 ± 1 (11 ± 1)	9 ± 1 (9 ± 1)	4 ± 0 (1)	3 ± 1
A3	5 ± 1 (0)	23 ± 1 (23 ± 1)	21 ± 1 (20 ± 1)	21 ± 1 (17 ± 1)	16 ± 1 (14 ± 1)	5 ± 1 (0)	5 ± 1
A4	7 (0)	16 ± 0 (11 ± 5)	18 ± 0 (18 ± 1)	24 ± 5 (23 ± 5)	31 ± 1 (29 ± 1)	14 (0)	1 ± 1
A5	14 ± 0 (0)	5 (5)	13 ± 0 (11 ± 0)	5 (5)	11 ± 0 (11 ± 0)	3 (0)	5 ± 1
A6	10 ± 1 (1)	28 ± 2 (17 ± 6)	20 ± 1 (20 ± 1)	11 ± 6 (11 ± 6)	15 ± 1 (13 ± 1)	10 ± 3 (1)	15 ± 3
D1	8 ± 1 (0)	29 ± 1 (27 ± 2)	17 ± 1 (17 ± 1)	33 ± 2 (32 ± 2)	22 ± 1 (22 ± 1)	13 ± 1 (0)	13 ± 2
D2	8 (0)	19 ± 0 (19 ± 1)	20 ± 1 (20 ± 1)	17 ± 1 (17 ± 1)	19 ± 1 (18 ± 1)	5 (0)	2 ± 1

The table also shows the number of units among these assigned to other muscles (in brackets), because the NMs model allows for one spike train to estimate the excitation of multiple muscles.

**Figure 2:**
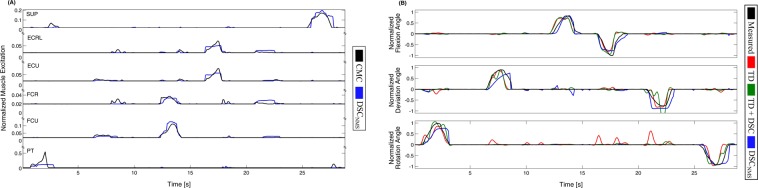
Representative example of the estimation results. (**A**) The muscle excitation estimations of the neuromuscular regression model compared to the excitations estimated by inverse kinematics and CMC. Note that in the CMC calculation non-agonist muscle excitations at increasing wrist angles are restricted to zero. (**B**) The kinematics estimation performance of the three methods (colored) compared to the measured kinematics (black).

The proposed NMS model based on the Decomposed Spike Count feature (DSC_NMS_) produced notably different kinematics estimations than the two other investigated approaches (Fig. 2). Most notably, DSC_NMS_ exhibited a smaller amount of spurious activations, smoother signal characteristics for increasing wrist angles, and more abrupt changes when returning to the rest position.

**Figure 3:**
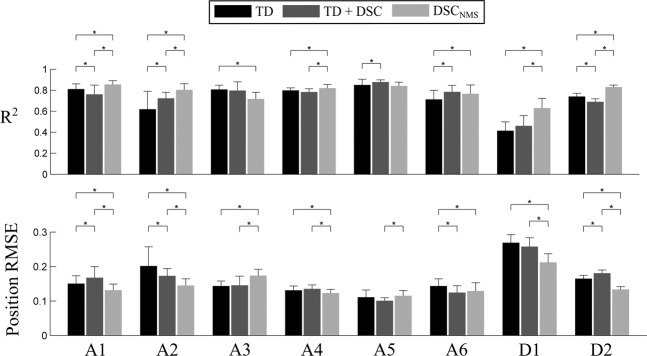
Regression accuracy of the investigated features for all subjects. The asterisks represent statistically significant differences. Note that for the R^2^, larger values correspond to better performance, whereas for the RMSE, smaller values are better.

The statistical analysis for the two considered performance metrics yielded similar results (Fig. 3), thus in the following we only report the analysis on the R2 value. showed no significant difference between groups (p = 0.06), and significant differences between features (p = 0.01) and subjects (p < 0.01), as well as a two-way interaction between subjects and features (p < 0.001). Therefore, we repeated the analysis for each subject individually, as described in Methods (Fig. 3). On average, the R^2^ value of the able-bodied subjects was 0.77 for Time Domain features (TD), 0.79 for Time Domain features combined with DSC (TD + DSC) and 0.8 for DSC_NMS_, corresponding to performance increases of 2.6% and 3.9% over TD respectively. For the transradial amputees, the mean R^2^ values were 0.58, 0.58 and 0.73, corresponding to a 25.9% increase in performance of DSC_NMS_ over TD (52.1% for D1 and 7.5% for D2).

The post-hoc tests showed that DSC_NMS_ outperformed TD in four of six intact-bodied subjects, and in both transradial amputees. For three subjects, there was no statistically significant difference between TD + DSC and DSC_NMS_, in the other cases, the proposed method outperformed TD + DSC significantly. TD + DSC outperformed TD in three subjects, there was no statistically significant difference in three cases including D1, and TD outperformed TD + DSC for A1 and D2.

## Discussion

We investigated an extension of neural regression with biomechanical modeling for myocontrol^24,25^. In this way, we reduced the complexity of the regression task by estimating muscle excitations instead of wrist kinematics directly. Then, we used a musculoskeletal model driven by the estimated muscle excitations for estimating the control output.

We observed that the proposed method outperformed state of the art Artificial Neural Network (ANN) regression in most intact-bodied subjects and in both transradial amputees tested. The NMS estimation was also more accurate than using an ANN with the TD + DSC feature set for most subjects, showing the benefit gained by the explicit definition of the musculoskeletal system in the control scheme.

The estimation results of the NMS model were not just more accurate (Fig. 3), but also steadier, with less sudden changes in direction or speed (Fig. 2). This might indicate that the superior performance of the NMS model was due to the smoothness of the estimate, in which case a simple low-pass filter could provide the same results. However, both TD and TD + DSC were low-pass filtered after estimation as well, meaning that in our experiments we were not able to improve the performance of pure ANN regression alone to the same level using low-pass filtering.

The relative performance of TD and TD + DSC might be explained by the difficulty of ANNs in processing missing spikes or spike trains in the same way mathematical viscoelastic muscle models do. We previously showed that in dynamic voluntary contractions, EMG decomposition might contain only a few spikes below 20% of the maximal angle in all DoFs and that most spike trains are not present in all repetitions of a given movement^12^. This was also confirmed in this dataset (Fig. 1, Tables 1 and 2). Notably, this missing information did not influence the performance of DSC_NMS_ in the same cases, due to the robustness of the neural regression sub-model (Section IV.D.2). This result indicates that despite the dependency of EMG decomposition accuracy on the thickness and type of subcutaneous tissue, the morphology of the muscles, training level, etc., a robust modeling scheme might be able to overcome many of those issues.

Although the performance gain for able-bodied subjects was only 3.9%, the gain for the transradial amputees was greater, especially for subject D2 (52.11%). This indicates that for able-bodied subjects the (nonlinear) mapping of EMG activity to wrist kinematics is less challenging, whereas at least for some transradial amputees, such a mapping can be substantially improved using motor-unit level information, for which case the NMS model might deliver better performance.

The number of spike trains decomposed with this method was comparable to decomposition results in isometric and non-isometric contractions^14,17,29,30^, with only a few decomposed spike trains in more than one DoF (Table 1). This was unexpected due to the nature of the tasks, which prompted the activation of the same muscles in different DoFs, e.g*. m. flexor carpi radialis* during flexion and radial deviation. Such common motor units might have been missed by the decomposition due to changes in the motor unit action potential waveform during voluntary contractions, meaning that EMG decomposition methods developed for such conditions might improve the performance of decomposition, and thus that of prosthesis control^17,31^.

The assignment of the decomposed spike trains to muscles resulted in either uniquely assigning them to either *musculus pronator teres* (PT) or *musculus suplinator* (SUP), or to multiple other muscles simultaneously (Table 2). This distribution can be attributed to the decomposition results. Since the same muscles were active during different DoFs, a unique identification was only possible for spike trains detected in both DoFs. Because this was only observed for a small number of spike trains, the unique assignment was not possible in most cases (Table 2). It has to be noted that physiologically both PT and SUP have flexion and deviation moments, but in our Computed Muscle Control (CMC) calculation, their contribution was negligible.

Despite the small number of uniquely identified spike trains, the NMS model provided an accurate feed-forward estimation of the muscle excitations (Fig. 3A). This estimation could also be useful for several other purposes than prosthesis control, such as diagnosing or monitoring muscle weakness after amputation or supporting the process of prosthesis training.

Based on the muscle excitations, the NMS model identified actuated DoFs (Fig. 3A). This allowed the elimination of unwanted activations of other DoFs, which were present for other methods (Fig. 3B), while maintaining the potential capability to estimate movements in multiple DoFs simultaneously – although such a scenario was out of scope for this study. The superior performance was achieved using a generic biomechanical model, without scaling it to the individual physiology of the subjects. Although subject-specific scaling of the models is crucial for general biomechanical modeling^22,32,33^, the scaling step is problematic for transradial amputees due to the missing limb. Our finding that a single general anthropometry was robust enough across subjects might indicate that for this application, the calibration of the model is not as critical as for other scenarios. This feature is an important potential benefit for clinical applications since long fitting and training times are associated with prosthesis abandonment^3^.

Prosthesis training could also be supported by biomechanical variables estimated by the NMS model, such as joint velocity or muscle excitation. Such knowledge could potentially reduce training time and facilitate a better understanding of prosthesis function by the patients, by showing the parameters to change during training to improve prosthesis control. This is a clear advantage of the NMS model over the low-level control schemes used in commercial prostheses, as well as over black-box machine learning approaches used in academia. In this context, the proposed scheme can be further extended and integrated with next-generation neuro-musculoskeletal modeling formulations based on the three-dimensional morphological representation of muscle fibers and series elastic tendons^33^.

There are two main limitations of this study. First, our approach is based on open-loop forward dynamics trained on a single task type and velocity, which might limit its generalization capability to other tasks, and might account for the robustness of the model even without scaling^28,34^. The aim of this proof of concept study was, however, to assess the feasibility and the performance of such an approach in simplified conditions. A thorough validation of such an approach would have to be performed online^35^, with real-time EMG decomposition methods for dynamic contractions^17^, which are not currently available, although there is considerable research in this direction^31,36^. While differences in offline scenarios might disappear in online testing^35^, the significant difference in performance for transradial amputees suggests that there is indeed a functional gain using NMS modeling, at least for some patients.

Second, the proposed model was not compared with state of the art EMG-based musculoskeletal models, which require targeted electrode placement over specific muscles^27,37^. Since EMG decomposition required high-density recordings, the assignment between channels and muscles was unknown, and additional targeted EMG placement was not feasible, especially for transradial amputees.

In conclusion, we showed that it is possible to outperform state of the art nonlinear regression using EMG features by using an NMS model driven by neural information decomposed from the surface EMG, in both intact-bodied subjects and transradial amputees, in an offline setting. The NMS regression method proposed in this study was able to accurately estimate muscle excitations during voluntary contractions and to track the kinematics more accurately than ANN regression, regardless of using TD features alone or together with neural information. This suggests that the increase in performance was not only due to neural information but also to the robust estimation of muscle excitations and biomechanical variables during movement. In addition, the proposed method might facilitate prosthesis training and the understanding of prosthesis function by patients. Our results indicate that using neural information for prosthesis control is a promising approach to provide a natural and intuitive control interface for patients with limb deficiency with application in the broad domain of wearable robotics^38^.

## Methods

**Figure 4:**
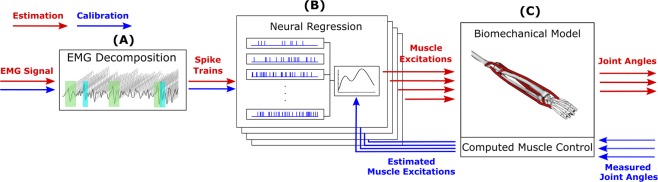
Block diagram of the proposed method. The input of the framework is the recorded multi-channel EMG signal, which is decomposed into motor unit spike trains (**A**). Features extracted from these trains are used to estimate muscle excitations (**B**), which are then used to simulate wrist kinematics using a biomechanical model (**C**). During the calibration phase (blue arrows), the biomechanical model is used in the inverse direction to compute the muscle excitations based on the recorded wrist kinematics. The computed muscle excitations are then used to calibrate the neural regression model (**B**). (**C**) has been obtained using the OpenSim software. OpenSim is an open-source software based on the Apache License, Version 2.0 (http://www.apache.org/licenses/LICENSE-2.0), which allows free publishing of OpenSim-generated figures and materials.

The control method proposed in this study consists of three steps (Fig. 4). First, motor unit action potential trains are decomposed from the recorded high-density EMG using convolutive blind source separation techniques. Then, a neural regression model is used to estimate muscle excitations based on the features extracted from the decomposed spike trains. The resulting muscle excitations are then used to simulate the wrist kinematics via a musculoskeletal model. This section describes each step of the method, the calibration of the used models, as well as the experiments used to calibrate and validate the method.

### Subjects

Six intact-bodied subjects (A1–6, 30 ± 6 years) and two transradial amputees (D1 and D2, 57/37 and 43/43 years/years-since-amputation respectively) participated in this study. Both amputees were daily prosthetic users. The University Medical Center Göttingen Ethical Committee approved all experimental procedures (Ethikkommission der Universitätsmedizin Göttingen, approval numbers 9/2/12 and 11/10/14) and all experimentation was performed in accordance with the relevant guidelines and regulations. All subjects signed an informed consent form.

### Data acquisition

We recorded surface EMG signals using high-density electrode grids (ELSCH064NM3, OT Bioelettronica) mounted around the residual limb, or for intact-bodied subjects around the proximal third of the forearm of the dominant hand (2 or 3 grids). The 8 × 8 grids had an inter-electrode distance of 1 cm. The electrode grids were connected to a 256-channel EMG amplifier (EMGUSB2, OT Bioelettronica). The recorded EMG signals were band-pass filtered between 3–900 Hz, and A/D converted using a 12-bit converter at a sampling rate of 2048 Hz.

To capture the joint angles of the wrist during the tasks and to provide visual feedback, three motion capture pods (MTx, Xsens) were applied on the palm, wrist and upper arm directly above the elbow, on the dorsal side for each location. For intact-bodied subjects, the motion capture pods were placed on the dominant arm, for transradial amputees on the contralateral arm.

### Experiment protocol

**Figure 5:**
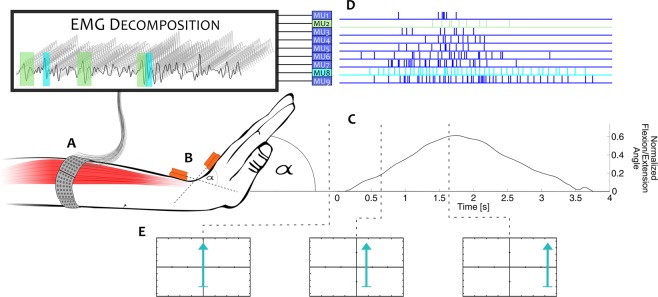
The recording setup, experimental protocol, and data processing. The EMG was recorded using high-density surface EMG (**A**) and the kinematics of the wrist were captured by motion capture pods (**B**). The subjects followed triangular joint angle profiles in three degrees of freedom separately (**C**). The recorded EMG was then decomposed (**D**) and used to drive a biomechanical model to estimate the recorded kinematics (**C**). The visual feedback shown to the subjects during the tasks was an arrow shaped cue (**E**), which they had to follow by controlling another arrow of the same size and different color with the movement of their wrist.

Subjects were seated in front of a monitor that provided visual feedback based on the captured intact wrist kinematics. First, subjects were asked to keep the arm fully relaxed at rest, and to position themselves on the chair so that the hand could move freely in any direction with the elbow relaxed, fully extended. During the recording, they were instructed to follow an arrow-shaped visual cue, which prompted movements along one DoF at a time. Horizontal movements of the arrow corresponded to flexion/extension, vertical movements to the ulnar/radial deviation, and rotation to pronation/supination. During the movement in each DoF separately, the wrist angle increased linearly from rest position to the maximum range of motion in 1 s, and then back in the same fashion after a 500 ms pause (Fig. 5).

Transradial amputees were also instructed to perform mirrored bilateral movements, matching the movement of the phantom limb to the contralateral side. For intact-bodied subjects mirrored movements were not needed as previous studies found no difference in myoelectric control performance between the ipsi- and contralateral sides in this group^27,32^. After a familiarization session and adjusting the range of the feedback to the subjects’ range of movement, each subject performed three repetitions of each direction of each DoF in a randomized order.

### Neuro-musculoskeletal mapping

Features were extracted from the decomposed spike trains, which served as the input signal of the control system. The framework consists of two sub-models (Fig. 4B,C): a neural regression model to estimate muscle excitations based on the extracted neural features, and a musculoskeletal model to estimate wrist kinematics from the estimated muscle excitations. In the following, we describe all three parts of the DSC_NMS_ framework.

#### EMG decomposition and feature extraction

The neural information was extracted from the high-density EMG signal using a decomposition method for convolutive mixtures^14^, as described in^29^. The silhouette (SIL) metrics was used as reliability measure for each individual motor unit spike train. Only motor unit spike trains with a SIL value higher than 0.8 were used for the analysis^14^.

The feature used as model input was the number of discharges of each decomposed spike train, calculated for each train within 100-ms observation sliding windows, with 10-ms overlap between consecutive windows. This feature set is referred to in this report as DSC.

#### Neural mapping

The discharge rate of a motor unit is determined by the net excitatory synaptic input (shared^39–41^ and independent) received by the motor neuron^41–43^. This relation was modeled for each spike train of a muscle by estimating the muscle excitation using the DSC as the input of a linear model with intercept. The ensemble muscle excitation was then calculated from the individual estimates by taking their median.

If there were no spike trains active in a given window, we used ANN regression to estimate the muscle excitations from TD features. This was done using one dedicated ANN for each muscle with the same parameters and training method as described in Section IV.E.2.

#### Musculoskeletal mapping

We used a generic musculoskeletal model of the arm available in OpenSim^44,45^. We reduced the model to the wrist joint and the following muscles acting on it: *musculus flexor carpi ulinaris* (FCU), *musculus flexor carpi radialis* (FCR), *musculus extensor carpi ulnaris* (ECU), *musculus extensor carpi radialis longus* (ECRL), *musculus pronator teres* (PT), *musculus supinator* (SUP).

In the muscle models, maximal isometric muscle forces were modified so that the numerical values of the muscle excitations were zero at the rest position of the wrist. This removal of the excitation offset was necessary because of the used muscle model, which cannot handle zero excitation values.

The excitations estimated by neural regression were used in an open-loop forward dynamics formulation to predict joint angles. For this purpose, excitations drove Hill-type muscle models to produce force profiles, which were projected onto a musculoskeletal model to produce joint moments and accelerations. Accelerations were then forward integrated to produce joint angular velocity and position for flexion/extension, ulnar/radial deviation, and pronation/supination. These single DoF joint angles variables constituted the output of the DSC_NMS_ framework.

Recent results showed that simultaneous and proportional control methods may have limited accuracy when controlling single DoF movements^46^. Thus, we refined the model to allow for precise single-DoF movements using the available biomechanical information, by only estimating a DoF if it was actuated by all (active) agonist muscles. For example, detecting activity in both FCU and FCR was interpreted as a flexion and only flexion/extension was estimated (the other DoFs were set to zero), whereas if any of the above muscles was active alone, both flexion/extension and radial/ulnar deviation were estimated.

#### Model calibration

Calibration of the model was necessary for two reasons. First, the recorded kinematics had to be scaled to the boundaries of the biomechanical model. This was needed because in a prosthesis control context, fitting the model parameters to physiological markers to account for differences in ranges of motion was not feasible due to the unknown parameters of the missing limb. The output of the biomechanical model was rescaled accordingly. Second, the neural regression models had to be trained for each decomposed spike train. For this purpose, we operated the biomechanical model using a closed-loop formulation (Fig. 4, blue arrows). We estimated muscle excitations from measured kinematics taken from a training dataset via CMC in OpenSim^47^. CMC tracks the experimentally recorded reference joint kinematics (Section II.C) by computing the underlying muscle excitations needed to drive the joint angles of the model. The muscle redundancy problem is solved using static optimization^3^.

To promote solutions that best fit the decomposed spike train patterns, we restricted antagonist muscle activations to zero when the absolute angle of the actuated DoF increased. This resulted in solutions with minimal co-excitation across agonist and antagonist muscle groups. The resulting muscle excitations were then used to train the neuromuscular regression model (Section IV.D.2), i.e. to establish a model between the input neural features and the excitations across all calibration trials (Fig. 2 in Results), in the following two steps.

The first step was to determine the location of the motor units in the different muscles. Indeed, since the neural information was obtained through blind source separation of the EMG, we could not assign the decomposed spike trains to a specific muscle-tendon unit in the model *a priori*. We assigned a motor unit to a muscle-tendon unit in the OpenSim musculoskeletal model, if the muscle excitation was greater than zero at the time of at least 80% of decomposed discharges. If this criterion corresponded to more than one muscle-tendon unit, the motor unit was assigned to all identified muscle-tendon units. Although this assignment did not reflect physiological conditions, it was necessary to populate all muscle-tendon units with spike trains in the redundant wrist-hand system.

In the second step, the relation between the firing statistics of individual spike trains and the muscle excitation was established. A robust linear estimator of muscle excitation was trained using the DSC of each spike train as input, for each observation window containing spikes. The linear fitting method employed iteratively reweighted least squares using the bisquare weight function^48^.

After calibration, the model operated in a purely open-loop way, i.e. without control mechanisms to compensate for kinematic drift. The feasibility of the approach was assured by the fact that CMC generates dynamically consistent excitation profiles. Because of this property, if the estimated excitations matched the ones generated by CMC during calibration, then the resulting model kinematics would match the profiles tracked by CMC, and therefore could be used in an open-loop forward dynamics formulation.

### Comparison with the state of the art

State of the art myoelectric control methods extract features from the interference EMG directly, and use machine learning techniques to estimate the joint angles continuously during movement. For comparison with our modelling approach, we implemented an ANN that is considered the currently best performing method according to^6,35^.

#### Feature extraction

The rectified EMG signal was digitally band pass filtered with a 4^th^ order Butterworth filter with cut-off frequencies 20 and 500 Hz^35^. In the same observation windows as used for the DSC feature set, we calculated the following TD features of the signal for each channel: root mean square, slope sign changes, zero crossings, and waveform length^5,49^. We then reduced the dimensionality of the data using principle component analysis (PCA) so that the resulting signal retained 98% of the original variance. The loadings were calculated using the whole training data, and the scores were extracted from the multichannel signal during estimation using these loadings.

#### Myoelectric regression

We implemented nonlinear regression with the neural network toolbox of MATLAB, using one separate network for each DoF and three neurons in one hidden layer per network, as suggested in^6,49^. The training was done using the Levenberg-Marquardt back-propagation algorithm. For each fold, we trained 50 ANNs using two repetitions of each DoF, and only the network with the best performance was used for further comparisons, as suggested in^50^.

To evaluate whether there was an increase in ANN regression performance due to including neural information in addition to the interference EMG features, we introduced a third feature set containing both the TD and the DSC features used as input for ANN regression. These were concatenated before the PCA, and then the same dimensionality reduction and regression training were applied as for TD.

### Statistical analysis

We used three-fold cross-validation to assess the regression performance for both the ANN and the NMS regression. From the three repetitions that the subjects performed, we randomly labeled two repetitions of each direction of each DoF as part of the training set. The testing set consisted of the remaining repetition of each direction of each DoF. The performance measures were the R^2^ value and the Root Mean Squared Error (RMSE) of the fitting between the estimated and the captured wrist kinematics. We repeated the cross-validation 10 times for both methods. For statistical analysis, we used an ANOVA model with the fixed factor “Feature” and the random factor “Subject”, which was nested within the fixed factor “Group” (with levels “Intact-Bodied” and “Transradial Amputees”). Individual analyses were carried out using one-way repeated measures ANOVA for each subject individually with the fixed factor “Feature”. Post-hoc tests were performed using the Bonferroni correction and significance was reported at p < 0.05. Means and standard deviations are reported. We performed the analysis on both performance measures separately.
